# Balancing to stay green: Two key proteins determine leaf color and photosynthesis in rice

**DOI:** 10.1093/plphys/kiaf670

**Published:** 2025-12-22

**Authors:** Gunjan Sharma

**Affiliations:** Assistant Features Editor, Plant Physiology, American Society of Plant Biologists; School of Biosciences, University of Birmingham, Edgbaston B15 2TT, United Kingdom

Chlorophyll is vital to carry out photosynthesis. Plants tightly regulate chlorophyll biosynthesis and catabolism through highly conserved pathways facilitating appropriate photosynthetic rate for growth and development including grain filling. The century-old source-sink theory suggests the importance of green leaves as a source of photosynthate production and mobilization to seed grains (Mason and Maskell 1928a, 1928b).

Rice is a major cereal crop, and understanding the molecular basis of grain size is imperative to address the challenges in tackling global food security. Four molecular pathways regulate seed grain size in rice: ubiquitin-proteasome, transcription regulatory factor, mitogen-activated protein kinase, and G-protein (Li et al. 2018). GRAIN WIDTH 2 (GW2) was the first E3 ligase identified as a regulator of grain size and weight. *gw2* mutants exhibited larger grain size, and it was shown that GW2 restricts spikelet (rice flower) cell proliferation (Song et al. 2007). GW2 targets multiple substrates, including a cell wall expansion protein EXPANSIN-LIKE 1 (EXPLA1), a glutaredoxin protein WIDE GRAIN 1 (WG1), and a C2H2 zinc-finger protein GRAIN WIDTH 9 (GW9) Abbas et al. (2026). GW2 also promotes leaf senescence and chlorophyll degradation (Shim et al. 2020). Intriguingly, GW2 has roles in developing flowers and has targets that affect photosynthesis. However, how GW2 regulates chlorophyll biosynthesis remains elusive.

In a recently published article in *Plant Physiology*, Ye et al. (2025) revealed the role of GW2-YLR (Yellow Leaf Rice) module in fine-tuning chlorophyll biosynthesis. The GW2 ring-type E3 ubiquitin ligase, co-localizes with the chlorophyll-biosynthesis enzyme YLR (ethylene reductase), ubiquitinating it and targeting it for 26S-proteasome degradation, thereby reducing chlorophyll production. YLR shares similarity to a divinyl reductase, known to catalyze divinyl chlorophyll *a* to monovinyl chlorophyll *a* (Wang et al. 2010).

The authors set out to reveal the downstream targets of *GW2* through EMS mutagenesis and suppressor screening of the *gw2* mutants in the rice *indica* variety. A mutant, *yellow leaf rice* (*ylr*) was identified to be supressing the dark green leaf color and high grain weight observed in *gw2*. The *ylr* mutants also exhibited pleiotropic growth defects such as retarded growth at the tillering stage. Chlorophyll measurements of the *ylr/gw2* double mutant revealed a reduction in total chlorophyll content (primarily chlorophyll *a*) and concomitant yellowing of leaves at the seedling and heading stage compared with wild-type (WT) and *gw2* leaves. The *ylr/gw2* mutants were significantly taller than *gw2* but shorter than WT. The seed weight and size of *ylr/gw2* mutants were less than *gw2*. However, seed number was reduced in both mutants compared with WT. This observation suggests that *YLR* counteracts the *GW2* function for increased chlorophyll and reduced height.

Using the fine-mapping approaches, the authors identified *LOC_Os03g22780* harboring a G-to-A nucleotide transition at base 631 substituting amino acid lysine for glutamine 211. This amino acid substitution disrupts the structure, impairing functionality. Complementation with the native *YLR* gene along with regulatory components rescued the *ylr/gw2* mutants back to green leaves. Furthermore, *YLR* expression was detected in leaves and stem tissue with low expression in roots.

YLR is involved in chlorophyll biosynthesis; therefore, the authors examined the ultrastructure of chloroplasts using transmission electron microscopy. The *ylr/gw2* chloroplasts showed a loose and disorganized grana morphology in contrast to tight grana stacks in *gw2.* The authors measured photosynthetic parameters and observed an overall reduction in photosynthesis rate caused by a reduction in photosynthetic efficiency via an altered electron transport chain, stomatal conductance, and transpiration rates. Overexpression of *YLR* restored the tight stacking of chloroplast grana and photosynthesis rates in *ylr/gw2* mutants. Overexpression and CRISPR-Cas 9 indels of *YLR* (independent of *gw2* mutant background) also showed similar effects, suggesting that *YLR* is a positive regulator of photosynthesis and maintains chloroplast ultrastructure and electron transport chain through chlorophyll biosynthesis. Furthermore, RNA-seq transcriptome analyses of mutants identified the differential regulation of photosynthetic and grain size development regulators between OE and *ylr* mutants. However, how GW2 regulated YLR function was yet unknown.

A transient expression of YLR exhibited cytoplasmic and chloroplast subcellular localization. Yeast 2-hybrid and co-immunoprecipitation showed that YLR and GW2 proteins interact in vitro and in vivo by transient bimolecular fluorescent complementation assay. In vitro E3 ubiquitin ligase assay confirmed that GW2 acts as an E3 ligase and targets YLR for ubiquitination.

In conclusion, Ye et al. (2025) shows that chlorophyll biosynthesis enzyme, YLR, is a target of the GW2 E3 ligase, finely tuning photosynthetic rates. However, imbalances such as *gw2* loss of function or *YLR* overexpression produced greener leaves, while YLR activity removal showed yellow leaves (Fig.). It is intriguing to uncover the novel targets of the GW2 E3 ligase to improve our understanding of grain development in rice. Commonly employed strategies of the yeast 2-hybrid system and target overexpression are ineffective for target identification, as GW2 self-activates resulting in false positive colonies and overexpression lines being lethal. Therefore, use of modern efficient strategies such as AlphaFold structural predictions coupled with biochemical assays for fishing out novel targets is indispensable for future investigations. Altering GW*2* or *YLR* expression reduces seed number. Investigating the role of GW2-YLR along with identifying the associate targets at the tissue- and cell-specific levels might improve rice grain filing.

**Figure.**
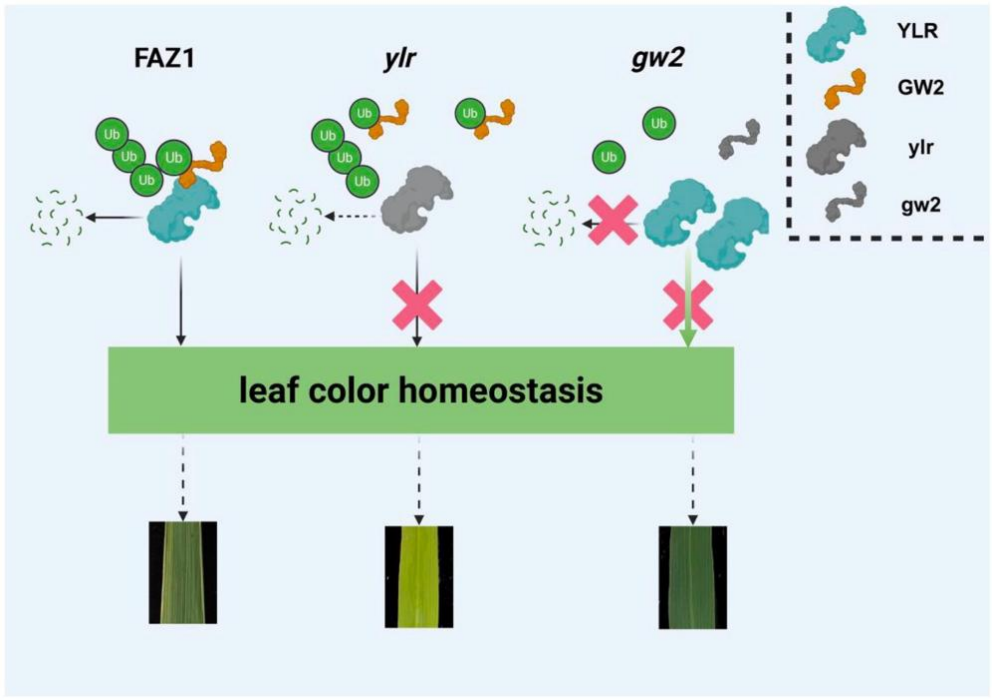
The GW2-YLR module fine-tunes molecular mechanisms and physiological processes governing the leaf greening trait for optimal photosynthetic rates. In WT *indica* rice variety FAZ1, GW2 maintains the green leaf color homeostasis by fine-tuning the accumulation of the YLR protein via polyubiquitination and 26S proteasome pathway-mediated degradation. The disruption of homeostasis by removal of GW2 in the *gw2* mutant mimics overexpression of YLR exhibiting green leaf, while *ylr* mutant results in yellow leaf color phenotypes and altered photosynthetic rates (adapted from Ye et al. 2025).

## Related articles published in *Plant Physiology*:

Bai et al. (2023) identified a natural variation in a gene regulating grain shapes in cultivated and wild rice varieties.

Fan et al. (2024) generated rice germplasm resources for future breeding of super high-yielding varieties with superior haplotypes in different target genes for tackling the challenge of food security and nutrition.

## Data Availability

No new data was generated or analyzed for this article.
